# Phytochemical Profile and Biological Activities of *Satureja hortensis* L.: A Review of the Last Decade

**DOI:** 10.3390/molecules23102458

**Published:** 2018-09-25

**Authors:** Irina Fierascu, Cristina Elena Dinu-Pirvu, Radu Claudiu Fierascu, Bruno Stefan Velescu, Valentina Anuta, Alina Ortan, Viorel Jinga

**Affiliations:** 1 National Institute for Research & Development in Chemistry and Petrochemistry-ICECHIM Bucharest, 202 Spl. Independentei, 060021 Bucharest, Romania; dumitriu.irina@yahoo.com; 2 University of Agronomic Sciences and Veterinary Medicine of Bucharest, 59 Mărăști Blvd., 011464 Bucharest, Romania; alina_ortan@hotmail.com; 3 University of Medicine and Pharmacy “Carol Davila”, 37 Dionisie Lupu Str., 030167 Bucharest, Romania; bruno_velescu@yahoo.co.uk (B.S.V.); valentina.anuta@umfcd.ro (V.A.); vioreljinga@yahoo.com (V.J.)

**Keywords:** *Satureja hortensis* L., antioxidant, antimicrobial, antiparasitic, pesticidal, anti-inflammatory, analgesic, hepatoprotective, anticancer

## Abstract

*Satureja hortensis* L. (summer savory) is an annual herbaceous crop, native to Europe and in our days spread and used all over the world. Although its use as spice and medicinal plant is known since ancient times, peer-reviewed studies presenting the scientific data are scarce. The natural products obtained from summer savory (extracts and essential oil) are dominated by polyphenols and flavonoids, responsible for their antioxidant, antimicrobial, antiparasitic, pesticidal, anti-inflammatory, analgesic, hepatoprotective and anticancer properties, among others. The current study presents the progress made in the last decade regarding the potential applications of summer savory, being the first review study focused on *S. hortensis*, in the same time suggesting future research opportunities, as they appear from the properties of other *Satureja* species. The available data presenting the properties of summer savory represents a scientific support for application in industry, for developing “clean label” food products.

## 1. Introduction

Summer savory (*Satureja hortensis* L.) represents an annual herbaceous crop species, strongly branched, with linear leaves belonging to the *Lamiaceae* family. The flowers, purple or violet white with red peeled on the inside, are grouped at the upper nodes of the branches [1]. As an aromatic plant, indigenous to southern Europe and Mediterranean area (in our days distributed across both hemispheres, in warmer regions and as a pot culinary herb), its aerial parts (especially the leaves) were traditionally used for culinary purposes and as a medicinal plant [2].

The main biomolecules found in extracts and essential oils of *S. hortensis* are the volatile oils, phenolic compounds, flavonoids, tannins, steroids, acids, gums, mucilage and pyrocatechols [3], leading to different potential applications in treating some very serious disorders, such as diabetes, cardiovascular diseases, cancer, Alzheimer’s, alongside the antioxidant, antimicrobial and anti-inflammatory properties.

**Figure 1:**
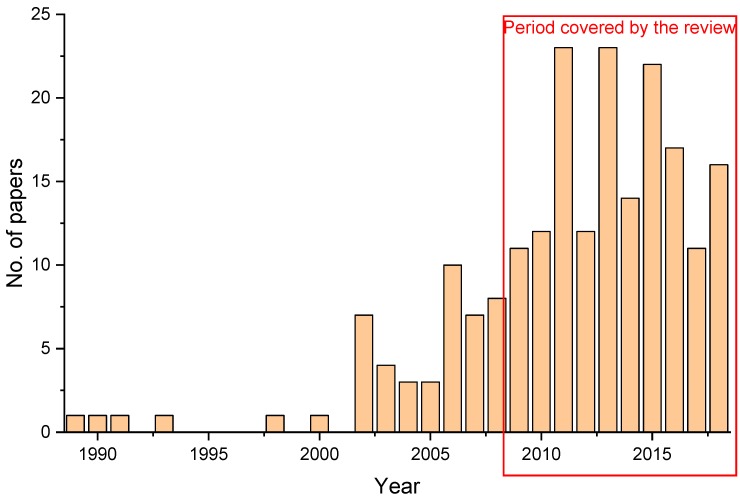
Distribution of publications per year from Scopus Database according to the keyword.

Even though the summer savory was listed as a medicinal herb since ancient times [4] and its’ traditional culinary uses are well-known (especially in the southern Europe and Mediterranean region), scientific literature presenting the potential biomedical applications of *S. hortensis* is surprisingly scarce. Thus, from the initial 381 documents returned by the search in SCOPUS database on the topic “*Satureja hortensis*”, only 209 documents spread over a period of 30 years (1989–2018) matched the keyword search “*Satureja hortensis effect*” (Figure 1). From the returned results, the focus of the present review was the last decade, leading to a final number of papers taken into consideration for the review of 161. In spite of the relatively few scientific papers published on this topic, the last decade has witnessed a very notable increase in the number of scientific studies regarding the properties and effects of *S. hortensis*.

The present review aims to present the progress made on the last decade regarding the potential application of biomolecules found in *S. hortensis*, in order to complete the review papers previously published [3,5,6,7] on the wider subject of *Satureja* species search: “*Satureja hortensis* effect” [8].

## 2. Composition of *Satureja hortensis* L.

The chemical composition of summer savory was revealed in several studies performed on *S. hortensis* volatile oils and, in fewer cases, extracts. Considering the general composition, the fresh leaves contain moisture (72%), protein (4.2%), fat (1.65%), sugar (4.45%), fibre (8.60%) and ash (2.11%) [2]. Discussing the composition on a dry weight basis, the main source for bioactive compounds is the volatile oil (up to 5%), triterpenic acids, tannins (up to 8%), mucilage, resins, sugars, mineral salts, etc. [1].

The volatile oil isolated from summer savory has as major components carvacrol, thymol, phenols, and flavonoids [6]. Several studies identified in the volatile oils as main components thymol (0.3–28.2%), γ-terpinene (15.30–39%), carvacrol (11–67%), and *p*-cymene (3.5–19.6%) [3,7,9,10,11,12]. Despite the obvious differences between their results, all studies generally present the same componence of the volatile oils (besides the major components identifying the presence of α-phellandrene, α- and β-pinene, sabinene, terpineol, α-thujene, etc.). The different results obtained by the authors are due to seasonal variation, climatic factors, agronomic procedure, genetic structure, etc. [13]. Thus, when discussing the potential application of essential oil or extracts obtained from summer savory, at least the harvesting area and general composition should be provided, in order to present a clear picture of the reviewed results.

The extracts obtained from aerial parts of *S. hortensis* represents the subject of significantly fewer studies, when compared with the essential oils. The methanolic extract obtained by maceration was proven to be dominated by rosmarinic acid (24.9 mg/g), caffeic acid (1.3 mg/g), naringenin (1.1 mg/g), isoferulic acid (220 µg/g) and apigenin (165 µg/g). Other flavones (luteolin) and their glycosides (apigetrin and vitexin), as well as flavonol (quercetin), flavonol glycosides (isoquercitrin, astragalin, quercitrin) and coumarin derivatives (aesculin and aesculetin) were also detected [14]. A comparative study on the effect of different extraction procedures (Soxhlet extraction, maceration, ultrasound-assisted extraction, microwave-assisted extraction, subcritical water extraction) on the composition of the summer savory extracts showed a variation of the total phenolic content between 119 and 151 mg gallic acid equivalents (GAE)/g, total flavonoids content between 5 and 28 mg rutin equivalents (RU)/g, condensed tannins between 41 and 73 mg GAE/g, gallotannins between 12 and 35 mg GAE/g and total anthocyanins content between 103 and 144 mg cyanidin-3-glucoside equivalents (CGE)/g, all superior for the subcritical water extraction. The HPLC quantification of several compounds revealed the presence of rosmarinic acid, quercetin, apigenin, kaempferol, luteolin, chlorogenic acid, rutin and apigenin-glycoside, among others, all molecules with demonstrated biological activities [15]. Rosmarinic acid and caffeic acid oligomers (clinopodic acids I, O and P) were also determined by Moghadam et al. [16] in methanolic extracts obtained using pressurized liquid extraction. The study of Mohtashami et al. [17] evaluated variation in composition of the summer savory essential oil under different storage conditions, focusing on the content in α-thujene, α-pinene, β-pinene, α-phellandrene, myrcene, α-terpinene, *p*-cymene, β-phellandrene, γ-terpinene and carvacrol. Their conclusions were that due to several mechanisms presented by the authors, the quality of the essential oil increased with storage time (expressed by the increase in carvacrol, *p*-cymene and β-phellandrene). The conclusion is of particular interest for industrial applications, in which the storage times are extensive.

The study of Estaji et al. [18] regarding the influence of salinity on several parameters of *S. hortensis* plants revealed that the phenolic compounds increased upon salinity stress, a non-enzymatic defense mechanisms against salinity-induced oxidative stress; a similar increase being observed for the essential oil quantity and quality (revealed by the increase in the content of main components: carvacrol, γ-terpinene, *p*-cymene, myrcene and β-pinene.

**Figure 2:**
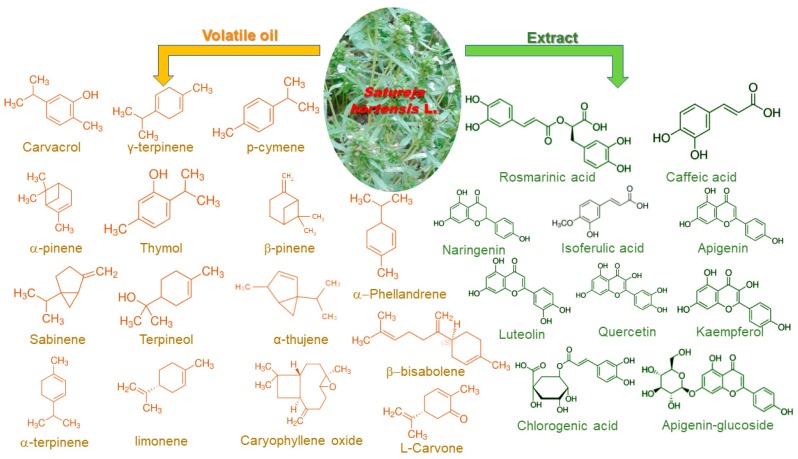
Main components of *S. hortensis* responsible for the biological activities.

Figure 2 summarizes the main components identified in the volatile oils and extracts of summer savory, while Table 1 summarizes the main findings regarding the composition of *S. hortensis*.

## 3. Biological Activities of *Satureja hortensis* L.

In the following paragraphs, the biological properties of summer savory will be discussed, based on examples from the relevant literature studies, completed with examples regarding the biological activities of pure compounds.

### 3.1. Antioxidant Properties

Due to the presence of polyphenolic compounds, *S. hortensis* essential oil presents antioxidant activity, demonstrated through in vitro studies [6]. Commercial essential oil (from Iran) was proven to exert a considerable antioxidant effect, as determined by different assays, such as 2,2′-diphenyl-1-picrylhydrazyl (DPPH), 2,2′-azinobis(3-ethylbenzothiazoline-6-sulfonate) diammonium salt (ABTS), ferric thiocyanate and β-carotene bleaching [19]. The addition of *Satureja hortensis* essential oil (SHEO—obtained by hydro-distillation from Turkish vegetal material) to chitosan nanoparticles offered the nanoparticles antioxidant properties (ranging from 43.66% to 56.99%, as determined by the DPPH assay), in direct correlation with the EO content [20]. A similar observation was made by Shojaee-Aliabadi et al. [21] upon the addition of Iranian commercial SHEO to κ-carrageenan films. On their study regarding the antioxidant potential of EO extracted from fifteen Iranian *S. hortensis* samples, Samadi et al. [22] obtained values of antioxidant activity index (determined by reporting the IC_50_ values obtained by the DPPH assay to the value resulted using the positive control BHT) between 0.17 and 0.46. The authors assign the antioxidant activity registered to the presence of *p*-cymene, carvacrol and β-bisabolene.

The extracts obtained from *S. hortensis* were also proven to have antioxidant properties: Mašković et al. [15] compared the antioxidant properties of extracts obtained by various methods from Serbian summer savory (methods presented in Section 2) using several assays: determination of total antioxidant capacity, lipid peroxidation assay, hydroxyl radical scavenging activity and DPPH assay. Their work demonstrated that the extract obtained by subcritical water extraction proved to be superior to the other methods in all the applied assays. The ethanolic extract of *S. hortensis* was evaluated for antioxidant properties, by comparison with *Artemisia dracunculus* extract [23]. Results of DPPH, ABTS and ferric reducing antioxidant power (FRAP) assays showed superior activity for the *S. hortensis* extract, in good correlation with the higher total phenolic and total flavonoid content registered.

The in vivo study published by Boroja et al. [14] revealed the ameliorating effects of methanolic extract of *S. hortensis* aerial part against cisplatin-induced oxidative damage in liver, kidney, and testes in rats, regulating levels of superoxide dismutase enzyme (SOD), catalase enzyme (CAT), glutathione (GSH) and thiobarbituric acid reactive substances (TBARS) affected by the administration of cisplatin.

Considering the main components identified in summer savory essential oil and literature survey, the SHEO antioxidant activity could be attributed to the high content in carvacrol, γ-terpinene, p-cymene and thymol compounds with known antioxidant activity [24,25]. In the same time, components of extracts of *S. hortensis* (rosmarinic acid, caffeic acid, naringenin, quercetin, apigenin, kaempferol, luteolin, chlorogenic acid, rutin and apigenin-glycoside) are also well-known for their antioxidant potential [26,27].

Due to the antioxidant potential shown, natural extracts obtained from *S. hortensis* are currently considered for use in meat industry (the water leaf extract increased the shelf-life of ground beef) [28] or as an antioxidant in mayonnaise formulations [29].

### 3.2. Antimicrobial Properties

The presence of monoterpenes such as carvacrol, cymene and thymol in the essential oil indicates the high possibility of antimicrobial activities against food, plants and human pathogens [30]. The evaluation of SHEO obtained from Iranian plants revealed a good antimicrobial activity against several types of microorganisms, with minimum inhibitory concentration (MIC) values ranging from 0.06 µL/mL for *Candida glabrata* to 8 µL/mL for *Pseudomonas aeruginosa* and minimal lethal concentration (MLC) values ranging from 0.06 µL/mL for *Candida glabrata* to 16 µL/mL for *Pseudomonas aeruginosa* [10]. The results were superior to those obtained for the control substances used (vancomycin, gentamicin and amphotericin) for all the studied lines, except *P. aeruginosa* [10]. The authors suggest that the antimicrobial activity of the EO could be attributed to its thymol and carvacrol content, with *p*-cymene indirectly increasing their effect. The antimicrobial mechanism is presented (damage in membrane integrity, causing leakage of ions and other cell compounds and eventually death), in the same time evaluating the individual EO’s components antimicrobial properties.

The addition of SHEO to κ-carrageenan films conferred on the materials antimicrobial properties against *S. aureus*, *E. coli*, *B. cereus*, *S. typhimurium* and *P. aeruginosa* (in decreasing order of the inhibition zone diameter), the films being more effective in direct contact [21]. The same observation was made by Feyzioglu and Tornuk [20] regarding the effect of chitosan nanoparticles loaded with SHEO on foodborne bacteria (*E. coli*, *L. monocytogenes* and *S. aureus*) obtaining positive results for materials having different EO content, in a concentration dependent manner. The authors avoid assigning the antimicrobial effect to a particular compound, but rather assign the effect to the synergistic effect of different components in the EO. The SHEO exhibited a notable inhibitory effect against several bacteria (*Bacillus cereus*, *Escherichia coli*, *Salmonella typhimurium*, *Staphylococcus aureus*) with controlled release when incorporated in alginate microparticles [31].

Spraying SHEO obtained from Iranian plants (major components—carvacrol, 54.1%, terpinolene, 20.6% and α-phellandrene, 5.3%) had a positive effect on the preservation of kiwi fruits in the post-harvest stage, by reducing fungal rot [32]. Belgian commercial SHEO exerted antimicrobial properties against bacteria commonly found on fishes—*L. innocua*, *P. fluorescens* and *A. hydrophila/caviae* (in disc diffusion experiments) and against *L. innocua* and *A. hydrophila/caviae* (in volatile diffusion experiments) [33].

SHEO was obtained by Dikbas et al. [34] from wild-growing Turkish plants, having as major components carvacrol (54.7%), γ-terpinene (20.9%), *p*-cymene (12.3%), α-terpinene (2.0%), and thymol (2.0%). The exposure to SHEO vapors reduced the decay of strawberry and grape fruit in post-harvest stage, especially at low temperatures (5 and 10 °C), in a dose-dependent manner. Similar results were obtained on grapes at post-harvest stage [35], as well as on fruits and vegetables post-harvest fungi. The effect of SHEO extracted from Iranian plants was observed on *Penicillium digitatum* and *Rhizopus stolonifer*) [36], *R. stolonifer*, *P. digitatum*, *Aspergillus niger* and *Botrytis cinerea* [37], *Aspergillus* species [38] and *Rhizopus stolonifera* [39]. The obtained results offer the possibility to develop natural antifungals for disease control in post-harvest stage.

Saharkhiz et al. [40] studied the effect of the plant different stages of development on the composition and antimicrobial properties of SHEO. The major component identified was γ-terpinene (except in ripened fruit stage where major component was carvacrol at 46.4%). The antimicrobial assays performed on several bacteria at concentrations between 0.03 and 8 µL/mL suggested promising potential for the development of antimicrobial agents.

SHEO obtained from Algerian plants tested on *Salmonella enterica* suggested the potential of *S. hortensis* natural products for the application in liquid whole eggs preservation [41], while the study regarding the effect of SHEO obtained from Turkish plants tested on *E. coli* inoculated on freshly cut cucumber and tomato suggested its potential application for fresh-cut product sanitation [42].

Essential oil obtained using an industrial method from Slovakian *S. hortensis* plants (dominated by carvacrol, γ-terpinene, α-terpinene and *p*-cymene) showed antimicrobial activity against 10 *Pseudomonas* species isolated from freshwater fish, with MIC values ranging from 6.25 µL/mL against *P. antarctica* to 25 µL/mL for *P. koreensis*, *P. mandelii* and *P. proteolytica* [43].

Evaluation of the antimicrobial potential of SHEO on human pathogens revealed a strong antimicrobial activity of oils obtained from Turkish plants against 15 periodontal pathogens [44] also exhibiting a limited anti-biofilm effect. Similar findings were reported by Sharifzadeh et al. [45] regarding the effect of Iranian SHEO on fifteen *Candida albicans* strains (the most common species of yeast responsible for oral candidiasis) isolated from HIV-positive patients, with MIC values ranging from 200 to 400 µL/mL.

Gumus [46] reported the effect of gamma-irradiation on the methanolic extracts obtained from three different spices collected from Turkey, including *S. hortensis.* The antifungal efficiency tested against two aflatoxigenic moulds *Aspergillus parasiticus*, revealed an increase with the exposure of the extract to low radiation doses (1.2 kGy), followed by the decrease with higher doses (3 and 5.1 kGy). The authors assign the decrease of the antifungal activity with the high irradiation doses to decreases or structural changes in the phenols, carvacrol and thymol contents of the irradiated extracts.

Kotan et al. [47] determined the antibacterial potential of EO and extracts (n-hexane, chloroform, acetone and methanol) obtained from Turkish *S. hortensis* against fourteen plant pathogenic bacteria by comparison with pure compounds (carvacrol and thymol). The EO showed a good antimicrobial activity against all tested strains (with MIC values ranging from 15.63 to 125 µL/mL), while the extracts had a weak activity against a limited number of pathogens. The pure compounds had MIC values ranging from 15.63 to 31.25 µL/mL. In the same time, the experiments performed on lettuce seeds showed that the hexane–methanol extract mixture can be used as a seed disinfectant and as potential control agents for management of bacterial diseases.

The extracts obtained by Mašković et al. [15] from Serbian plants using different techniques extraction techniques showed antimicrobial efficiency against fifteen non-pathogenic or facultative pathogenic bacteria, with MIC values ranging from 7.81 to 250.00 μg/mL.

### 3.3. Antiparasitic and Pesticidal Properties

Although several plants from the *Satureja* family possesses antiparasitic properties [30], the literature regarding such effects of *S. hortensis* is very scarce. Hezarjaribi et al. [48] (citing Safarnejad Tameshkel et al. [49]) presents the antiparasitic effect of Iranian *S. hortensis* alcoholic extracts on *Giardia* cysts, with a mortality of 84.3%. Similar results were obtained by Soosaraei et al. [50], citing Pirali et al. [51] regarding the application of *S. hortensis* natural products as an antiparasitic agent against *Leishmania major*.

The SHEO obtained by hydrodistillation using plants collected from the Czech Republic (with a composition dominated by carvacrol and γ-terpinene) was studied for larvicidal activity against *Culex quinquefasciatus* Say (Diptera: Culicidae). From the study, covering twenty-two aromatic plant species, resulted that the SHEO had a good larvicidal activity (LC_50_ –*lethal concentration 50*-of 36.1 µg/mL), with a total mortality of 75% and an 100% deterrence of female oviposition for a concentration of 0.02% EO [52].

SHEO obtained from Turkish vegetal material (characterized by GC-MS as dominated by carvacrol, γ-terpinene, *p*-cymene and thymol) was evaluated as a natural insecticide against broadbean weevil (*Bruchus dentipes*) [53]. The results suggested a good insecticidal activity, with an 83.3% mortality at a concentration of 5 µL/L and an exposure time of 6 h. The mortality increased with both the concentration of EO and exposure time, reaching a 100% mortality at a concentration of 20 µL/L and a contact time of 24 h. By discussing the differences observed between the insecticidal activity of *S. hortensis* and *Origanum acutidens* correlated with their composition, the authors assign the toxic effect to carvacrol, in the same dime drawing attention to the potential synergistic and antagonistic effects of other compounds.

Yildirim et al. [54], evaluated the fumigant activity against adults of *Sitophilus granaries* of eleven plant species from Turkey, at four different concentration, obtaining 31.00–33.00% mortality, after 24 h, at concentrations between 1–20 μL oil.

Sajfrtova et al. [55] studied the insecticidal effect of some Lamiaceae extracts and essential oils obtained by several methods from plants growing in the Czech Republic. The extracts and the EO (dominated in composition by carvacrol, γ-terpinene and *p*-cymene) were tested in terms of acute toxicity, by measuring the mortality after 24 h, by application to the larvae of *Spodoptera littoralis*, *Musca domestica*, *Culex quinquefasciatus*, and *Leptinotarsa decemlineata* and to the adults of *M. domestica* and *L. decemlineata*. Summer savory natural products (especially the EO and CO_2_ extract) were active against all tested insects, the most sensitive being the larvae of Colorado potato beetle, with a LD_50_ of 22 µg.

Essential oil obtained from *S. hortensis* Iranian plants (with major constituents carvacrol, γ-terpinene, thymol, α-pinene and β-ocimene) was successfully encapsulated in chitosan/TPP (*tripolyphosphate*) nanoparticles by Ahmadi et al. [56]. Having the advantage of a controlled release (established over a period of 600 h, thus offering a sustained release of the EO), the materials proposed showed a very good acaricidal activity against adults and eggs of *Tetranychus urticae* Koch. The results indicated that encapsulation enhanced the SHEO fumigant toxicity after 24 h with the LC50 values significantly higher than the pure EO. This approach increased the longevity of lethal activity, with a mortality of 82% after 14 days, compared with the lethality of pure SHEO, reduced to 12% after the same time period.

The potential application of *S. hortensis* essential oil as a natural herbicide was recently presented by Hazrati et al. [57]. The SHEO obtained by hydrodistillation from aerial parts of plants collected at the beginning of the fruit stage was dominated by carvacrol and γ-terpinene (55.66% and 31.98%, respectively, determined by GC-MS). The EO, formulated as nanoemulsion (EO concentration 5 mL/L) exhibited herbicidal activity against *Amaranthus retroflexus* and *Chenopodium album* (at a nanoemulsion concentration of 1 mL/L in laboratory and 4 mL/L in greenhouse conditions), two weeds with world-wide spread.

### 3.4. Anti-Inflammatory and Antinociceptive Properties

Inflammation represents a defence appearing as a response to pathophysiological problems [3]. The search of new, more powerful anti-inflammatory drugs and analgesics represents an important area of research, with many works being focused on natural alternatives [6]. In this context, *S. hortensis* represents the subject of several studies presenting its anti-inflammatory and antinociceptive potential.

Hajhashemi et al. [58] presented the antinociceptive and anti-inflammatory effects of several natural products obtained from Iranian *S. hortensis* roots (essential oil, hydroalcoholic extract and polyphenolic extract). Using acetic acid-induced writhing test, formalin licking test and carrageenan-induced paw edema, the authors observed the inhibition of the abdominal writhes of all products, reduction of the licking time in the acute formalin test by the hydroalcoholic extract and by all materials in the chronic phase, as well as the anti-inflammatory potential of the EO (400 µL/kg), hydroalcoholic extract (100 and 200 mg/kg) and polyphenolic extract (400 mg/kg). Vafaei et al. [59] obtained good results using hydroalcoholic extract for diminishing the morphine withdrawal syndrome signs.

The review paper of Bahmani et al. [60] presents the antinociceptive effect of aqueous extract of *S. hortensis* determined by formalin and tail flick test; the anti-inflammatory potential of summer savory are also presented by other review papers [3,5,6,61].

### 3.5. Hepatoprotective Properties

The hepatoprotective role of *S. hortensis* extract was presented by Boroja et al. [14] for treating cisplatin-induced liver injury in rats, in correlation with the antioxidant properties (see Chapter 3.1), obtaining a maximum dose safe to apply under 200 mg/kg b.w. The results using *S. hortensis* extract were similar regarding the regulation of the hepatic serum parameters similar to silymarin (a known natural hepatoprotective drug). Although good anti-diabetic and anticholesterolemic properties were reported for other *Satureja* species [3,6,30], there were no scientific studies found regarding those effects of *S. hortensis* products in the time range reviewed. This could be an opportunity for future studies, as the EO and extracts are rich in carvacrol and thymol (responsible for lowering the serum cholesterol levels) and in flavonoids (possessing antioxidant and anti-hyperlipidemic properties) [3].

### 3.6. Anticancer Properties

The study of Misharina et al. [61] presented effect of SHEO intake on mice with high cancer risk. By the intensification of polyunsaturated fatty acids synthesis and reduction of lipid peroxidation products, the intake seems to be beneficial. The essential oil delivered through the drinking water (0.15 mg/L, daily intake approx. 2–3 mL) over a period of ten months, led to a significant increase of saturated and polyunsaturated fatty acids and a decrease of monounsaturated acids in brain and liver, accompanied by a reduction of lipid peroxidation products, thus proposing SHEO for therapeutic and preventive purposes. Evaluation of Canadian summer savory for antimutagenic potential [62] showed a neutral character for both the extract and of metabolites from herb extracts, with an antimutagenity of 8.15 and respectively 33% at 3360 µg/mL. The lack of literature data is surprising, as several others *Satureja* species exhibited anticancer potential: *S. intermedia* EO on oesophageal squamous cell carcinoma and human bladder carcinoma cell lines, *S. spicigera* on HT29/219 (*Rectosigmoid adenocarcinoma cells*), Caco2 (*human epithelial colorectal adenocarcinoma cells*), NIH-3T3 (*mouse embryo fibroblast cell*) and T47D (*ductal carcinoma cells*) lines, *S. sahendica* EO on MCF7 (*breast cancer cells*), Vero (*fibroblast-like kidney cells*), SW480 (*colon adenocarcinoma cells*) and JET 3 (*choriocarcinoma cells*) lines, *S. montana* on HT29 (*colon adenocarcinoma cells*) line, etc. [3,30].

### 3.7. Other Biological Properties

Ceker et al. [63] studied the effects of SHEO against aflatoxin B1 (AFB1) in human lymphocytes in vitro. The results suggested strong antioxidative and antigenotoxic effects, expressed by the decrease of sister chromatid exchange and micronuclei, as well as malondialdehyde, superoxide dismutase and glutathione peroxidase levels, increased as a result of the AFB1 action.

Several review papers from the last decade present other activities of *S. hortensis* natural products such as antiviral properties (evaluated against HIV virus) [6,64,65], inhibitory effects of methanol extracts on adhesion of the activated human platelet to laminin-coated plates [3,30], antispasmodic and diuretic [27], anti-asthenic, anti-dysenteric, bronchidilatator and carminative [66], expectorant [67], emmenagogue, aphrodisiac and resolutive [68] or for stomach pain [69].

The muscle relaxant properties of *S. hortensis* on tracheal smooth muscles contracted by methacholine and KCl were reviewed by Shakeri et al. [70], the relaxant properties being attributed to the presence of carvacrol.

Although the original research articles do not match our search criteria (studies published in the last decade), we consider that the properties should be mentioned, to provide a better image regarding the potential applications of this understudied source.

Other potential applications remain to be studied, considering the known effects of other *Satureja* species, such as anti-Alzheimer disease agent, anti-leishmanial, anti-protozoal, trypanocidal, etc. [71,72].

Table 2 summarizes the main biological activities, as emerging from the literature survey, considering the main constituents of the tested products.

## 4. Dosage, Toxicology, Popular and Emerging Uses

Summer savory represents an aromatic plant known and safely-used since ancient times. For example, evidences regarding *S. hortensis* use were discovered in a Roman colony in Northern Italy established in 183 BC [73]. When discussing dosage and toxicology, we must consider the main applications of the summer savory: spice, medicinal plant and essential oil.

As spice, the distinctive taste of summer savory led to its world-wide use and cultivation. The leaves are used as seasoning in stuffing and meat dishes, especially in Europe, while the sprigs can be boiled with peas or cabbage for improving digestion [2]. It is one of the ingredients of *Herbes de Provence* (together with rosemary, thyme and oregano); the extensive use as a spice, with no reported harmful effects suggests that summer savory it is safe for consumption as a spice.

As a medicinal plant, summer savory can be consumed either as hot tea or cold infusion and, more recently, in tablet form. As infusion, it can be prepared from two teaspoons of herb to a cup of water, 2–3 cups a day [1]. As a remedy for high blood pressure, the recommended cure comprises of one tablet containing 250 mg dried leaves for sixty days [3]. With very rare side-effects, savory products must be administered with caution by diabetics and people with hypoglycemia, high blood pressure or bleeding disorders and are not recommended to children and pregnant women [1,3,74].

Summer savory essential oil is considered safe for human consumption by the U.S. Food and Drug Administration [75]. As every essential oil, SHEO can produce dermal irritations and of the mucous membranes [1,76]. Pure SHEO has an oral of 1370 mg/kg (rats) and a dermal toxicology of 340 mg/kg (guinea pig) [77], Buckle [78] estimating a 19 mL oral lethal dose for a child. Due to its particular scent, SHEO found application in perfumery industry [79] and beverage industry (for the production of vermouth) [80], besides the classical application in food industry or in commercial spice mixtures for sausages, pâtés or pickles [63].

More recently, based on the properties presented in the previous chapters, natural products obtained from *S. hortensis* found application in meat industry [28], food antimicrobials [81], food packaging [21] and fresh vegetables preservation [82]. Thus, due to its high content in antimicrobial and antioxidant compounds (such as carvacrol, *p*-cymene and thymol), SHEO could offer an alternative for increasing the shelf life of meat products or vegetables [81,82,83,84]. Although the essential oil in general (and SHEO in particular) are extensively studied for different activities, due to their characteristics (volatility, thermal decomposition, unstable nature), they should be incorporated in different matrixes for topical applications, as presented in previous chapters [20,21,31,56,57,82,83,84].

More recently, *S. hortensis* leaf extract found application in nanotechnology. Using aqueous leaf extracts obtained from plants cultured under different salinity conditions, Rasaee et al. [85] phytosynthesized silver nanoparticles with diameters around 3 nm, observing a reduction of nanoparticle size with the increase in salinity [85]. This was most probably due to the increase in phenolic compounds observed by other authors upon salinity-related stress [18]. The obtained nanoparticles showed antimicrobial activity against *Bacillus subtilis*, *Bacillus vallismortis* and *Escherichia coli* [85].

## 5. Conclusions

Summer savory (*Satureja hortensis* L.) is known and used since ancient time as herbal remedy, spice and food flavoring. Original from Europe, it is now grown and used in most parts of the world, in several industries. With a composition dominated by polyphenols and flavonoids, the natural products obtained from summer savory were proven to have antioxidant, antimicrobial, antiparasitic, pesticidal, anti-inflammatory, analgesic, hepatoprotective and anticancer properties, among others. The literature survey demonstrates that the chemical composition of the natural products is changed significantly with the cultivation and geographical conditions, as well as with the stages of development at harvesting time.

The current study aimed to present the progress made in the study of this understudied plant in the last decade. According to our findings, most of the literature data focuses on antioxidant and antimicrobial potentials of *S. hortensis*, although the different uses demonstrated for other *Satureja* species would suggest the exploration of new applications. This, in turn, would be a promising research area for future studies.

Exploration of new application could result in the development of new natural alternatives for serious illnesses, such as cancer, Alzheimer, cardiovascular diseases and many others. In the same time, application of already established properties at industry scale would provide alternatives for “clean label” food products.

## Figures and Tables

**Table 1:** Phytochemical profile of *S. hortensis* as presented by original research papers.

Origin	Harvesting	Material	Composition (Main Components)	Analysis Method	Ref.
Serbia, cultivated	Beginning of flowering stage	Aerial parts—Essential oil	Carvacrol (67.00%), γ-terpinene (15.30%), *p*-cymene (6.73%), α-terpinene (1.29%), β-caryophyllene (1.90%) β-bisabolene (1.01%)	GC-FID, GC-MS	[9]
Iran, Cultivated	-	Aerial parts—Essential oil	Thymol (28.2%), *p*-cymene (19.6%), γ-terpinene (16%), carvacrol (11%), β-pinene (4.5%), sabinene (4.4%), α-pinene (2.7%), 4-terpineole (1.6%)	GC-MS	[10]
Lorestan province, Iran, wild-growing	Summer	Aerial parts—Essential oil	Carvacrol (48%), γ-terpinene (24.2%), *p*-cymene (11.7%), α-thujene (2.3%), α-pinene (2.5%), myrcene (2.5%), β-pinene (1.6%)	GC-FID and GC-MS	[11]
Isparta, Turkey, cultivated	Beginning of flowering stage	Aerial parts—essential oil	Carvacrol (50.5%), γ-terpinene (32.7%), *p*-cymene (3.4%), α-terpinene (4.1%), β–myrcene (2.1%) α–thujene (2.1%), α-pinene (1.5%)	GC and GC-MS	[13]
Kütahya, Turkey, cultivated	Beginning of flowering stage	Aerial parts—essential oil	Carvacrol (46.9%), γ-terpinene (38.7%), *p*-cymene (3.6%), α-terpinene (3.8%), β–myrcene (1.9%) α–thujene (1.3%), α-pinene (0.7%)	GC and GC-MS	[13]
Eskişehir, Turkey, cultivated	Beginning of flowering stage	Aerial parts—essential oil	Carvacrol (47.7%), γ-terpinene (34.5%), *p*-cymene (4.0%), α-terpinene (4.1%), β-myrcene (2.4%) α-thujene (2.1%), α-pinene (1.6%)	GC and GC-MS	[13]
Bursa, Turkey, cultivated	Beginning of flowering stage	Aerial parts—essential oil	Carvacrol (42.3%), γ-terpinene (36.7%), *p*-cymene (4.0%), α-terpinene (4.9%), β-myrcene (2.8%) α-thujene (2.9%), α-pinene (2.2%)	GC and GC-MS	[13]
Tokat, Turkey, cultivated	Beginning of flowering stage	Aerial parts—essential oil	Carvacrol (41.4%), γ-terpinene (36.6%), *p*-cymene (5.5%), α–terpinene (4.7%), β-myrcene (2.7%) α-thujene (2.5%), α-pinene (2.3%)	GC and GC-MS	[13]
Serbia, wild-growing	Flowering season (August)	Aerial parts—methanolic extract	Rosmarinic acid (2.49%), caffeic acid (0.129%), naringenin (0.106%), isoferulic acid (0.022%), apigenin (0.016%)	UHPLC	[14]
Serbia, wild-growing	August	Aerial parts—Soxhlet extraction, ethanol	TPC: 119.28 mg GAE/g, TFC: 5.23 mg RU/g, CT: 41.74 mg GAE/g, GA: 12.32 mg GAE/g, TAC: 103 mg CGE/g, rosmarinic acid 301 µg/g, quercetin 155 µg/g, luteolin 40 µg/g, kaempferol 46 µg/g, apigenin 52 µg/g, chlorogenic acid 36 µg/g, rutin 33 µg/g, apigenin-glycoside 24 µg/g	Colori-metric, HPLC	[15]
Serbia, wild-growing	August	Aerial parts—Maceration-ethanol	TPC: 125.34 mg GAE/g, TFC: 16.27 mg RU/g, CT: 47.2 mg GAE/g, GA: 18.54 mg GAE/g, TAC: 115.21 mg CGE/g, rosmarinic acid 287 µg/g, quercetin 1.7 µg/g, luteolin 1.2 µg/g, kaempferol 11 µg/g, apigenin 3 µg/g, chlorogenic acid 17 µg/g, rutin 10 µg/g, apigenin-glycoside 2 µg/g	Colori-metric, HPLC	[15]
Serbia, wild-growing	August	Aerial parts—Ultrasounds extraction-ethanol	TPC: 132.4 mg GAE/g, TFC: 19.68 mg RU/g, CT: 52.65 mg GAE/g, GA: 21.87 mg GAE/g, TAC: 121.59 mg CGE/g, rosmarinic acid 1.3 µg/g, quercetin 6.4 µg/g, luteolin 0.8 µg/g, kaempferol 1.2 µg/g, apigenin 1.4 µg/g, rutin 24 µg/g, apigenin-glycoside 0.8 µg/g	Colori-metric, HPLC	[15]
Serbia, wild-growing	August	Aerial parts—Microwave extraction-ethanol	TPC: 147.21 mg GAE/g, TFC: 23.1 mg RU/g, CT: 64.43 mg GAE/g, GA: 25.35 mg GAE/g, TAC: 135.32 mg CGE/g, rosmarinic acid 9.6 µg/g, quercetin 41.2 µg/g, luteolin 1.1 µg/g, kaempferol 1.9 µg/g, apigenin 2.3 µg/g, rutin 28.4 µg/g, apigenin-glycoside 2.6 µg/g	Colori-metric, HPLC	[15]
Serbia, wild-growing	August	Aerial parts—Subcritical water extraction	TPC: 151.54 mg GAE/g, TFC: 28.2 mg RU/g, CT: 73.2 mg GAE/g, GA: 31.5 mg GAE/g, TAC: 144.57 mg CGE/g rosmarinic acid 2.6 µg/g, quercetin 11 µg/g, luteolin 0.4 µg/g, kaempferol 1.1 µg/g, apigenin 0.8 µg/g, rutin 16.6 µg/g, apigenin-glycoside 0.88 µg/g	Colori-metric, HPLC	[15]
Switzerland, Cultivated	-	Aerial parts—methanolic extract	Rosmarinic acid 4.2 mg/g, clinopodic acid i 1.8 mg/g, clinopodic acid O—1.1 mg/g, clinopodic acid P—0.5 mg/g	HPLC	[16]
Iran, cultivated	Full flowering stage	Aerial part—essential oil	α-Pinene (0.81%), α-thujene (1.3%), α-phellandrene (0.33%), β-pinene (0.45%), α-terpinene (3.79%), myrcene (2.05%), β-phellandrene (0.26%), *p*-cymene (2.53%), γ-terpinene (35.4%), carvacrol (50.69%)	GC-MS	[17]
Iran, cultivated	Flowering stage	Leaves—ethanol extract	0.26–0.32 mg/g, depending on the accession	Colori-metric	[18]
Iran, cultivated	Flowering stage	Aerial part—essential oil	Carvacrol (26–45.6%), γ-terpinene (14.9–22.33%), *p*-cymene (9.84–32.31%), myrcene (2.23–2.78%), β-pinene (1.20–1.73%) depending on the accession	GC-MS	[18]

Where GC-FI—Gas Chromatography-Flame Ionization Detector, GC-MS-Gas—chromatography–mass spectrometry, (U)HPLC—(ultra-)high-performance liquid chromatography, TPC—Total phenolic content, TFC–Total flavonoids content, CT—Condensed tannins, GA—Gallotannins, TAC—Total anthocyanins content, GAE—gallic acid equivalents, RU—rutin equivalents, CGE–cyanidin-3-glucoside equivalents.

**Table 2:** *Satureja hortensis* L.—natural products and biological activities presented in the original research papers reviewed.

Origin	Part of Plant/Product	Activity	Tests Performed	Main involved Components	Ref.
Iran, cultivated	Aerial parts/EO	Antimicrobial	Micro broth dilution assay.	Thymol, carvacrol, *p*-cymene, other minor components	[10]
Serbia, wild-growing	Aerial parts/extract	Antioxidant, Hepatoprotective	*In vivo* assay–determination of SOD, CAT, GSH and TBARS/biochemical analyses, histopathological analyses	Rosmarinic and caffeic acids	[14]
Serbia, wild-growing	Aerial parts/extract	Antioxidant, Cytotoxic, Antibacterial	Total antioxidant capacity, lipid peroxidation, hydroxyl radical scavenging, DPPH/MTT assay/MIC determination by microdilution method	Total phenolics, total flavonoids, condensed tannins, gallotannins, total anthocyanins	[15]
Iran, commercially available	EO	Antioxidant	DPPH, ABTS, Ferric thiocyanate, β-carotene bleaching, Tiobarbituric acid assays	Total phenolic compounds: 293.7 mg GAE/mL EO	[19]
Turkey, Cultivated	Leaves/EO loaded in chitosan nanoparticles	Antioxidant, Antimicrobial	DPPH assay, Broth dilution method	Carvacrol	[20]
Iran, commercially available	EO in κ-carrageenan films	Antioxidant, Antimicrobial	DPPH assay, Disc diffusion method, Disc volatilization method	Carvacrol, γ-terpinene and *p*-cymene	[21]
Iran, wild-growing	Aerial parts, EO	Antioxidant	DPPH assay	*p*-Cymene, carvacrol β-bisabolene	[22]
Iran, wild-growing	Aerial parts, extract	Antioxidant	DPPH, ABTS, FRAP assays	Total phenolic content, Total flavonoid content	[23]
Turkey	Leaves/extract	Antioxidant, Antimicrobial	TBARS assay/Total aerobic mesophilic, psychrotrophic, Pseudomonas and enterobacteriaceae counts	NA	[28]
Iran, commercially available	EO	Antioxidant	Peroxide value, Conjugated diene hydroperoxides, Thiobarbituric acid value	NA	[29]
Iran, commercially available	EO loaded in chitosan microparticles	Antioxidant, Antimicrobial	DPPH assay/Agar diffusion assay	Carvacrol, γ-terpinene, *p*-cymene	[31]
Iran, wild-growing	Aerial parts/EO	Antifungal	Evaluation of the decay of treated kiwi fruits	Thymol, other minor components	[32]
Belgium, commercially available	EO and EO-containing biopolymers	Antimicrobial	Disc diffusion method, Vapor-phase antimicrobial activity, MIC determination	Phenolic compounds	[33]
Turkey, wild growing	Aerial parts/EO	Antifungal	Evaluation of the treated fruits decay	Carvacrol and thymol	[34]
Iran, wild growing	Aerial parts/EO	Antifungal	In vitro antifungal test, Evaluation of the treated grapes decay	Phenolic compounds	[35]
Iran, cultivated	Aerial parts/EO	Antifungal	Poison food medium and vapor phase assay methods	Phenolic components (thymol, carvacrol), synergetic effects	[36]
Iran, wild-growing	Aerial parts/EO	Antifungal	Determination of MIC and MFC, Evaluation of the treated strawberry decay	Carvacrol, thymol	[37]
Iran, cultivated	Leaves and flowers/EO	Antifungal	Poisonous medium technique	Phenolic compounds	[38]
Iran	EO	Antifungal	Agar dilution method, vapor phase assay	Phenolic compounds (thymol and carvacrol)	[39]
Iran	Aerial parts/EO	Antimicrobial	Broth dilution method	γ-Terpinene, carvacrol	[40]
Algeria, wild-growing	Aerial parts/EO	Antibacterial	Agar diffusion, Determination of MIC, liquid whole eggs inoculated with *S. enteritidis* and exposed to EO	Synergetic effects between major components (carvacrol, *p*-cymene, γ-terpinene) and minor constituents	[41]
Turkey, cultivated	Leaves/hydrosol	Antimicrobial	Inhibition effect on *E. coli* inoculated tomato and cucumber	Carvacrol, thymol, *o*-cymene, linalool, borneol	[42]
Slovakia, commercially available	EO	Antioxidant, Antimicrobial	DPPH assay/Agar disc diffusion method, detection of MIC	Carvacrol, γ-terpinene, α-terpinene, *p*-cymene	[43]
Turkey, wild-growing	Leaves and flowers/EO	Antimicrobial	Determination of MIC, effect on biofilm formation	Carvacrol	[44]
Iran, cultivated	Leaves/EO	Antimicrobial	Microdilution broth susceptibility method, determination of MIC and MFC, antibiofilm–MTT reduction assay	Thymol, γ-terpinene, carvacrol, *p*-cymene	[45]
Turkey, wild-growing	Flowers and leaves/Extracts	Antifungal	Inhibition of fungal growth, effect on mycelium weight	NA	[46]
Turkey, wild-growing	Aerial parts/EO, extracts	Antibacterial/germination inhibition	Disc diffusion method, MIC determination/Pot assay	Carvacrol and thymol	[47]
Iran	Leaves/extract	Antiparasitic	Evaluation of cysts fatality placed near extract	NA	[48,49]
Iran	Aerial parts/EO	Antiparasitic	Evaluation of *Leishmania* parasites survival after exposure to EO	NA	[50,51]
Czech Republic, wild-growing	Aerial parts/EO	Larvicidal	Mosquito larvicidal assay, effect of lethal doses on larval development, oviposition deterrent effect	Carvacrol, γ-terpinene, *p*-cymene, α-terpinene, myrcene, β-bisabolene	[52]
Turkey, wild-growing	Aerial parts/EO	Insecticidal	Evaluation of mortality of *B. dentipes* exposed to EO	Carvacrol, thymol, *p*-cymene	[53]
Turkey, wild-growing	Aerial parts/EO	Insecticidal	Evaluation of the mortality rate of adults of *S. granaries* exposed to EO	NA	[54]
Czech Republic, cultivated	Aerial parts/EO, extracts	Insecticidal	Mortality determined by topical application to *Spodoptera littoralis*, *Musca domestica*, *Culex quinquefasciatus*, *Leptinotarsa decemlineata*	Carvacrol, γ-terpinene, *p*-cymene/volatile components in isolates	[55]
Iran, wild-growing	Aerial parts/EO	Acaricidal	Evaluation of fumigant toxicity against adults and eggs of *Tetranychus urticae* Koch	Monoterpenes and monoterpene hydrocarbons	[56]
Iran, wild-growing	Aerial parts/EO	Herbicidal/germination inhibition	Evaluation of weed control properties/evaluation of germination percentage upon exposure to EO nanoemulsion	Carvacrol, γ-terpinene, minor components	[57]
Iran, wild-growing	Seeds/EO, extract, polyphenolic fraction	Antinociceptive, Anti-inflammatory	Mice and rats tests-Acetic acid-induced writhing, Formalin test/carrageenan-induced rat paw edema	γ-Terpinene, thymol/flavonoids, polyphenoli ccompounds	[58]
Iran	Aerial parts/extract	Detoxification	Diminishing the morphine withdrawal syndrome signs	NA	[59]
Great Britain, commercially available	EO	Anticancer, Chemopreventive	*In vivo* assay on mice with cancer risk; biochemical analyses, histopathological analyses	NA	[61]
Canada, cultivated	Aerial parts/Extract	Antimutagenic	*Umu* test	NA	[62]
Turkey, wild-growing	Aerial parts/EO	Protective effect against AFB_1_ mutagen, Antioxidant	Measurement of SCE and MN frequencies, biochemical analyses (SOD, GPx, MDA).	Carvacrol, thymol, α-terpinene, γ-terpinene, *p*-cymene	[63]

Where: EO—essential oil, GAE—gallic acid equivalents, FRAP-Ferric reducing antioxidant power, SOD–Superoxide dismutase enzyme, CAT–Catalase enzyme, GSH—Glutathione, TBARS–Thiobarbituric acid reactive substances, MIC—Minimum inhibitory concentration, MFC—minimum fungicidal concentration, SCE—sister chromatid exchange, MN–micronucleus, GPx–glutathione peroxidase, MDA—malondialdehyde, NA—not available (not provided by authors).
